# Pre-clerkship National Board of Medical Examiners Subject Examinations Versus End-of-Semester Final Examinations: How Well Do They Assess Preparedness for the United States Medical Licensing Examination Step 1?

**DOI:** 10.7759/cureus.30523

**Published:** 2022-10-20

**Authors:** Marvi Moreno, Pedro Gonzalez, Blake Sieck, Edward Simanton

**Affiliations:** 1 Educational Outcomes and Assessment, Kirk Kerkorian School of Medicine at University of Nevada, Las Vegas, Las Vegas, USA

**Keywords:** usmle, assessment, medical education, licensure examination, exam performance

## Abstract

Background

The ability to provide performance insights of various United States Medical Licensing Examination (USMLE) Step 1 assessments is of great importance to medical educators. Two custom pre-clerkship assessments used at the Kirk Kerkorian School of Medicine at the University of Nevada, Las Vegas (KSOM) are National Board of Medical Examiners (NBME)-derived end-of-semester final examinations and subject examinations. The authors sought to determine if performance on these custom assessments can provide feedback on a medical student’s readiness to undertake the USMLE Step 1 examination.

Methodology

Deidentified student performance data were provided by institutional databases for the KSOM graduating class of 2023 (N = 60). Pearson correlation analyses were utilized to evaluate the strength of the correlation between USMLE Step 1 performance and NBME subject examinations versus NBME end-of-semester final examinations.

Results

The results indicated that the NBME end-of-semester final examinations have a statistically higher correlation to the USMLE Step 1 score than the majority of the individual NBME subject examinations. However, the mean NBME subject examination score (Semester 1: r = 0.53, p < 0.05; Semester 2: r = 0.58, p < 0.05) demonstrated significantly higher correlation to the USMLE Step 1 performance than the NBME end-of-semester final examination score for both Semesters 1 and 2 (Semester 1: r = 0.50, p < 0.05; Semester 2: r = 0.48, p < 0.05).

Conclusions

These results showed that the mean of the NBME subject examination score was a better metric to assess readiness for the USMLE Step 1 than the NBME end-of-semester final examinations. However, each NBME end-of-semester final examination score showed a better correlation than the majority of the NBME subject examinations.

## Introduction

Medical schools prioritize methods in which they can guide and assess their students for success [1]. Objective determination of a medical student’s success involves considering a multitude of metrics and attributes throughout their medical training [2]. For example, one large factor for students attempting to match into residency with the National Residency Matching Program (NRMP) after medical school has been the United States Medical Licensing Examination (USMLE) Step 1 [3,4]. In recent years, the USMLE Step 1 score has increasingly been used as a screening tool for residency applicant selection because it is a nationally recognized metric that allows residency programs to compare applicants from different medical schools [5]. As the USMLE Step 1 is changing from a three-digit scored examination to a pass/fail examination, its use during the residency match process in the coming years will most likely change [6].

While successful completion of USMLE Step 1 is multifactorial, the aim of this study is focused on pre-clerkship examination metrics and their value in providing feedback to students as they navigate their pre-clerkship medical school years. Determining indicators of success on the USMLE Step 1 is of great importance because this information would allow interventions to be provided to address identified deficiencies [7,8]. Addressing the deficiencies would also ultimately increase the likelihood of at-risk students obtaining a favorable USMLE Step 1 outcome [9].

The Kirk Kerkorian School of Medicine at the University of Nevada, Las Vegas (KSOM), which has an 18-month pre-clerkship curriculum that spans three semesters, uses various performance assessments. At the KSOM, the following three pre-clerkship performance assessments are used: (a) National Board of Medical Examiners (NBME) subject examinations, (b) NBME end-of-semester final examinations, and (c) Comprehensive Basic Sciences Examination (CBSE). The NBME subject examinations and end-of-semester final examinations are assessments that the KSOM faculties derive from the NBME item bank. In contrast, the CBSE is a standardized practice examination developed by the NBME for the USMLE Step 1 that many schools administer [10].

While the NBME-customized subject examinations and CBSE are relatively common in medical education, the end-of-semester final examinations are not widely used [11]. Several studies have suggested that cumulative exams could potentially improve a student’s overall learning and retention. Studies conducted on university psychology and mathematics students who took cumulative examinations found that these students retained information longer than those who took non-cumulative final examinations [12-14]. Cumulative exams are important because they help students with the retrieval of information accumulated throughout the semester [15]. Additionally, students who were expecting a cumulative final examination may continue to process the learned information allowing it to be more readily accessible [16].

Between the NBME subject examinations and NBME end-of-semester final examinations, we seek to identify which of these custom assessments provides better feedback in assessing content mastery and overall preparedness of the students for the USMLE Step 1. We hypothesize that NBME end-of-semester final examinations would provide better insight regarding USMLE Step 1 performance than the NBME subject examinations.

## Materials and methods

**Figure 1:**
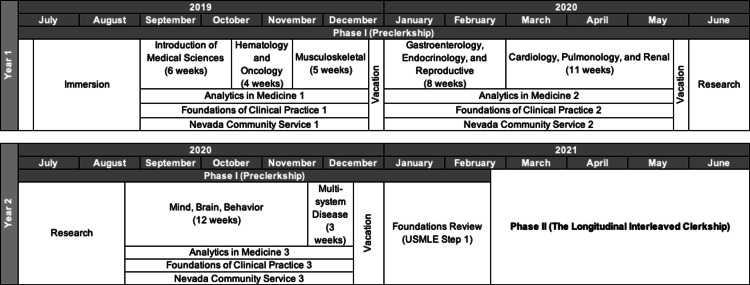
Schematic of the first two years of the Doctorate of Medicine program at the Kirk Kerkorian School of Medicine at the University of Nevada, Las Vegas. USMLE: United States Medical Licensing Examination

Deidentified student performance data and demographic information were provided by institutional databases with an approved Institutional Review Board (IRB) protocol. The first two years of the pre-clerkship curriculum were the focus of this study (Figure 1). The data came from the KSOM examination scores recorded by course directors for the graduating class of 2023 during their pre-clerkship coursework. The curriculum is broken down into system blocks ranging from three to 12 weeks. Every two to four weeks, a 50-75-item customized multiple-choice examination derived from the NBME item bank corresponding to each system was administered.

At the end of the first two pre-clerkship semesters, a 60-item NBME end-of-semester final examination was administered that comprehensively covered the content of all organ systems covered within a semester. The customized examination was built with an evenly distributed number of faculty-selected NBME questions for each system to allow for individual evaluation. An overall passing threshold of 70% was required for every student, as well as a passing threshold of 70% for individual systems covered on the final examination for students who needed to remediate the section. To successfully progress through the curriculum, students were required to meet the 70% passing thresholds for all NBME subject examinations as well. The CBSE is administered to conclude the end of the third semester.

A Pearson correlation analysis was performed to investigate the relationship between NBME end-of-semester final examination scores and USMLE Step 1 performance, as well as the NBME subject examination scores and USMLE Step 1 performance. Statistical significance was set at p-value ≤0.05. All calculations were done using SPSS version 26.0 (IBM Corp., Armonk, NY, USA). This study was approved by the KSOM Biomedical Institutional Review Board (approval number: 1030906-1).

## Results

Data from 60 students in the class of 2023 were collected. Presented in Table 1 are the demographic and background characteristics of the students included in this study. The students were divided relatively evenly by gender (52% male and 48% female). The average age for this cohort was 24 years old. Of the 60 students, 47% were Caucasian, 53% were considered underrepresented in medicine, 37% indicated that they were disadvantaged, and 30% were first-generation college students.

**Table 1:** Kirk Kerkorian School of Medicine at the University of Nevada, Las Vegas demographic characteristics of 60 students from the class of 2023.

Characteristics	N (%)
Total number of students	60
Gender	
Men (%)	31 (52%)
Women	29 (48%)
Mean age (years)	24
Ethnicity	
American Indian or Alaska Native	0 (0%)
Asian	21 (35%)
Black or African American	2 (3%)
Hispanic, Latino, or of Spanish Origin	8 (13%)
Native Hawaiian or Other Pacific Islander	0 (0%)
White	28 (47%)
Other	6 (10%)
Underrepresented in medicine	
Aggregate total of African Americans and/or Black, Hispanic/Latino, Native American (American Indians, Alaska Natives, and Native Hawaiians), Pacific Islander, and mainland Puerto Rican	10 (17%)
Aggregate total of African Americans and/or Black, Hispanic/Latino, Native American (American Indians, Alaska Natives, and Native Hawaiians), Pacific Islander, and mainland Puerto Rican Plus those matriculants who considered themselves disadvantaged	32 (53%)
Disadvantaged	
Yes	22 (37%)
No	38 (63%)
First-generation	
Yes	18 (30%)
No	42 (70%)

A Pearson correlation analysis was performed to examine the relationship between the examinations taken by the class of 2023 during the pre-clerkship phase at the KSOM (Table 2). Although students were required to take the NBME system examinations, end-of-semester final examinations, and CBSE examinations, a small number of excused reasons (health reasons and unforeseen life events) occurred. Thus, a completed set of data for all NBME system examinations, NBME end-of-semester final examinations, and CBSE were available for 57 out of 60 students (95%) in the class of 2023.

**Table 2:** Pearson correlation analysis between USMLE Step 1 and pre-clerkship examination metrics from 57 students, KSOM class of 2023. USMLE: United States Medical Licensure Examination; CBSE: Comprehensive Basic Science Exam Notes: * p-value <0.05. ** p-value <0.01.

Course – Exam	Topics	USMLE Step 1	
		Pearson Correlation	Significance (two-tailed)
USMLE Step 1		1	
Introduction to Medical Sciences – Exam 1	Cell biology, biochemistry, and molecular biology	0.302*	0.022
Introduction to Medical Sciences – Exam 2	Genetics, pathology, pharmacology	0.491**	<0.001
Introduction to Medical Sciences – Exam 3	Microbiology, Immunology	0.365**	0.005
Hematology and Oncology – Exam 1	Blood and the lymphatic system	0.343**	0.009
Hematology and Oncology – Exam 2	Hematology and related pathologies	0.381**	0.003
Musculoskeletal and Skin – Exam 1	Musculoskeletal system and related pathologies	0.059	0.665
Musculoskeletal and Skin – Exam 2	Musculoskeletal and integumentary systems and related pathologies Dermatology	0.355**	0.007
Mean Semester 1 (Systems)		0.527**	<0.001
Semester 1 (Comprehensive)		0.504**	<0.001
Gastroenterology, Endocrine, and Reproductive – Exam 1	Gastrointestinal system and related pathologies	0.321*	0.015
Gastroenterology, Endocrine, and Reproductive – Exam 2	Gastrointestinal system and related pathologies	0.393**	0.003
Gastroenterology, Endocrine, and Reproductive – Exam 3	Endocrine system and male reproductive system	0.560**	<0.001
Gastroenterology, Endocrine, and Reproductive – Exam 4	Reproductive system and pregnancy	0.261	0.054
Cardiovascular, Pulmonary, and Renal – Exam 1	Cardiovascular system	0.272*	0.043
Cardiovascular, Pulmonary, and Renal – Exam 2	Cardiovascular system and related pathologies	0.176	0.191
Cardiovascular, Pulmonary, and Renal – Exam 3	Renal system and related pathologies	0.434**	0.001
Cardiovascular, Pulmonary, and Renal – Exam 4	Pulmonary system and related pathologies	0.416**	0.001
Mean Semester 2 (Systems)		0.582**	<0.001
Semester 2 (Comprehensive)		0.475**	<0.001
Mind, Brain, and Behavior – Exam 1	Neuroanatomy	0.174	0.197
Mind, Brain, and Behavior – Exam 2	Neurology and special senses	0.300*	0.023
Mind, Brain, and Behavior – Exam 3	Behavioral health and sciences	0.157	0.244
Mean Semester 3 (Systems)		0.316*	0.017
CBSE		0.824**	<0.001
Mean (Systems)		0.662**	<0.001
Total (Comprehensive)		0.718**	<0.001

The significantly correlated NBME subject examinations demonstrated a correlation of 0.272 to 0.560 to USMLE Step 1 examination performance. The following examinations revealed no significance: Musculoskeletal and Skin - Exam 1 (r = 0.059), Gastroenterology, Endocrine, and Reproductive - Exam 4 (r = 0.261), Cardiovascular, Pulmonary, and Renal - Exam 2 (r = 0.176), Mind, Brain, and Behavior - Exam 1 (r = 0.174) and Mind, Brain, and Behaviour - Exam 3 (r = 0.157).

The mean NBME subject examination scores for each semester showed significant correlation ranging from 0.316 to 0.582 to the USMLE Step 1 examination was observed for Semester 1 (p < 0.000), Semester 2 (p < 0.001), and Semester 3 (p < 0.017). The mean of all the NBME subject examinations taken was calculated, and a correlation of 0.662 to the USMLE Step 1 performance was identified (p < 0.001).

Semester 1 NBME end-of-semester final examination showed a correlation of 0.504 (p < 0.001), while Semester 2 revealed a correlation of 0.475 (p < 0.001). Both NBME end-of-semester final examinations demonstrated a lower correlation than the mean of the NBME subject examinations at the end of each semester.

At the end of Semester 3, a CBSE assessment that addressed all the objectives throughout the pre-clerkship curriculum was administered to the class of 2023 medical students. A correlation (r) of 0.824 (p < 0.001) was found between the CBSE and USMLE Step 1. The mean of all the examinations including the CBSE has a correlation (r) of 0.718 (p < 0.001). This is higher than what was observed for the mean NBME subject examinations.

## Discussion

Results obtained from the analyses indicate that the NBME subject examinations administered during the pre-clerkship phase of the medical education curriculum at the KSOM demonstrated a varying but mostly significant correlation to the scores on the USMLE Step 1 examination. The correlation between the NBME system examinations and the USMLE Step 1 examination provides some degree of validation of the NBME subject examinations as an assessment tool for a student’s medical knowledge. Additionally, the overall performance on these subject examinations across all systems during the pre-clerkship curriculum provided a better insight into a student’s overall content mastery and preparedness to take USMLE Step 1.

On the other hand, the NBME end-of-semester final examinations administered at the end of each semester are another method used by KSOM to assess the academic aptitude of their medical students. Due to the similarities in item format and content scope, the CBSE has been established as a standard method of assessing a medical student’s success in the USMLE Step 1 examination [9]. Our data showed that the CBSE shows the greatest correlation to the USMLE Step 1, which is consistent with the conclusions of previous studies [2].

Both NBME end-of-semester final examinations administered at the end of the first and second semesters also demonstrated a significant correlation to USMLE Step 1 scores. However, these correlations are lower for the corresponding mean NBME subject examinations for the same semester. These findings are not in congruence with our hypothesis that the NBME end-of-semester final examinations will provide better feedback regarding USMLE Step 1 performance than the NBME subject examinations.

One potential explanation for this outcome is that the mean NBME system examinations have a better potential in capturing competency about a system than the end-of-semester final examination. This result may be due to the higher aggregate number of questions taken on all the NBME system examinations throughout the semester. Additionally, the NBME end-of-semester final examinations are secondarily used as a remediation tool for students who failed one or more of the NBME system examinations. Students who have successfully passed all the NBME subject examinations within the semester might be inclined to prepare only to obtain a passing score. Lastly, the preparation time for the NBME end-of-semester final examinations is significantly less in comparison to each NBME system examination.

We initially inferred that the NBME end-of-semester examinations would show a more meaningful relationship with USMLE Step 1 performance. With the expectation of being tested regarding the same concept at different levels during the pre-clerkship curriculum, with the USMLE Step 1 as the ultimate step, we also expected that this outcome would result in improved retention and recall of the learned information. This idea is further supported in that initial tests enhance long-term retention and final test performance [16]. However, our data demonstrated a different outcome. From a study that obtained comparable results, the authors hypothesized that the benefits of cumulative examinations might be less observed on higher reasoners [17]. Additionally, the highest improvement in the recall of material was mainly observed in low-scoring students [18]. This finding suggests that the characteristics of students enrolled in a program might affect the effectiveness of the end-of-semester final examination as an assessment tool.

In a survey of medical schools, the end-of-semester final examination is an assessment method not widely utilized by medical schools within the United States [11]. The results obtained from our analyses can help programs gain insight when deciding whether to augment their curriculum with this assessment tool in gauging the preparedness of their students to take the USMLE Step 1. From the results of our analysis, we infer that a student’s performance across all NBME subject examinations is superior to the NBME end-of-semester final examination in providing feedback regarding a student’s readiness to take the USMLE Step 1 examination. Moreover, a history of poor outcomes on an NBME subject examination might indicate a deficiency that needs to be addressed prior to undertaking the USMLE Step 1 to increase the likelihood of passing [19]. The current study supports that the NBME subject examinations in combination with the CBSE performance are crucial indicators for a successful outcome in the USMLE Step 1.

We recognize several limitations within our study. First, the interpreted data was derived from a small sample size and the scope of the study covered a single cohort at a single medical school. Furthermore, the student demographics for the study sample included over 50% of students who identify as non-white, 17% ethnically underrepresented in medicine, 30% first generation, and 37% economically disadvantaged. This range shows the large diverse population at KSOM, which could impact the generalizability as it relates to the demographics at other medical schools. Second, the curriculum structure is not consistent across all medical schools. KSOM endorses an accelerated program that prepares students to enter dedicated USMLE Step 1 preparation after 18 months and requires them to take the USMLE Step 1 by the end of Year 2, which is earlier than most schools.

Further validation of the data we provided could be granted through multi-cohort and multi-institutional analyses to compare results. It will also be important to analyze future metrics to determine how effective these correlations are across time and for clinical performance. Meaningful information could be gathered by following cohorts through the completion of the medical school curriculum and obtaining information on shelf examination scores, USMLE Step 2 performance, and residency program matching. This information could be compared across institutions to examine how pass/fail curriculums compare to graded curriculums. Other future studies should look to identify other variables that could give further insight regarding USMLE Step 1 performance, such as subgroup analyses on different student backgrounds.

## Conclusions

This study aimed to identify the most appropriate examination metric to utilize when assessing a medical student’s preparedness for the USMLE Step 1 during the pre-clinical curriculum. Although significant correlations were observed between the different forms of assessments and USMLE Step 1, performance on the NBME subject examinations provided the most insight regarding a student’s readiness to undertake USMLE Step 1. Identifying substandard performance in any of these subject examinations necessitates early interventions to maximize the student’s likelihood of obtaining a passing USMLE Step 1 outcome.
